# Supplementary material for: Trends in Clinico-Epidemiological Profile and Outcomes of Patients with HIV-Associated Cryptococcal Meningitis in Shanghai, China, 2013–2023
Source: Viruses. 2024 Aug 21;16(8):1333. doi: 10.3390/v16081333 (PMC11359124; doi:10.3390/v16081333)
Supplement: Supplementary file 1 [file viruses-16-01333-s001.zip › viruses-3089431-supplementary.pdf]

Table S1: Other characters of patients with HIV-associated cryptococcal meningitis

Parameters	Years											Subtotal	Spearman	*P*-Value
	**2013**	**2014**	**2015**	**2016**	**2017**	**2018**	**2019**	**2020**	**2021**	**2022**	**2023**			
Confirmed cases in HIV/AIDS patients														
CM	26 (9.32%)	29 (10.39%)	31 (11.11%)	37 (13.26%)	28 (10.04%)	29 (10.39%)	23 (8.24%)	30 (10.75%)	26 (9.32%)	13 (4.66%)	7 (2.51%)	279	-0.039	<.001†***
NCM	629 (6.71%)	778 (8.3%)	836 (8.91%)	908 (9.68%)	1043 (11.12%)	973 (10.37%)	1014 (10.81%)	869 (9.27%)	1044 (11.13%)	672 (7.16%)	613 (6.54%)	9379		
Cases of deceased														
Death	4 (13.79%)	3 (10.34%)	4 (13.79%)	5 (17.24%)	2 (6.9%)	2 (6.9%)	2 (6.9%)	4 (13.79%)	2 (6.9%)	1 (3.45%)	0 (0%)	29	-0.066	0.266†
Survival	22 (8.8%)	26 (10.4%)	27 (10.8%)	32 (12.8%)	26 (10.4%)	27 (10.8%)	21 (8.4%)	26 (10.4%)	24 (9.6%)	12 (4.8%)	7 (2.8%)	250		

Data are expressed as n (%) or median (Q1-Q3). Results are based on non-empty rows and columns in each innermost sub-table. †. Significance value of Trend Chi-Square test. ‡. Significance value of Kruskal-Wallis non-parametric test. *. significant at the .05 level. **. significant at the .01 level. ***. significant at the .001 level. CM: cryptococcal meningitis. NCM: not cryptococcal meningitis. CSF: cerebrospinal fluid.

Table S2: Initial clinical presentation of patients with HIV-associated cryptococcal meningitis

**Parameters**	**Years**											**Subtotal**	**Spearman**	***P*-Value**
	**2013**	**2014**	**2015**	**2016**	**2017**	**2018**	**2019**	**2020**	**2021**	**2022**	**2023**			
Cases Number	**26 (9.32%)**	**29 (10.39%)**	**31 (11.11%)**	**37 (13.26%)**	**28 (10.04%)**	**29 (10.39%)**	**23 (8.24%)**	**30 (10.75%)**	**26 (9.32%)**	**13 (4.66%)**	**7 (2.51%)**	**279**	-	-
Fever	19 (10.92%)	16 (9.2%)	16 (9.2%)	17 (9.77%)	14 (8.05%)	20 (11.49%)	18 (10.34%)	23 (13.22%)	20 (11.49%)	8 (4.6%)	3 (1.72%)	174	0.100	$0.115^{\dagger}$
Headache	19 (11.05%)	18 (10.47%)	21 (12.21%)	22 (12.79%)	18 (10.47%)	20 (11.63%)	18 (10.47%)	14 (8.14%)	14 (8.14%)	5 (2.91%)	3 (1.74%)	172	-0.123	$0.032^{\dagger*}$
Fatigue	12 (9.76%)	13 (10.57%)	11 (8.94%)	14 (11.38%)	10 (8.13%)	12 (9.76%)	11 (8.94%)	16 (13.01%)	15 (12.2%)	6 (4.88%)	3 (2.44%)	123	0.074	$0.212^{\dagger}$
Poor appetite	11 (9.91%)	10 (9.01%)	12 (10.81%)	10 (9.01%)	10 (9.01%)	13 (11.71%)	9 (8.11%)	15 (13.51%)	14 (12.61%)	6 (5.41%)	1 (0.9%)	111	0.071	$0.271^{\dagger}$
Vomiting	11 (10.48%)	12 (11.43%)	11 (10.48%)	12 (11.43%)	4 (3.81%)	13 (12.38%)	8 (7.62%)	10 (9.52%)	13 (12.38%)	7 (6.67%)	4 (3.81%)	105	0.053	$0.309^{\dagger}$
Weight loss	13 (17.81%)	6 (8.22%)	12 (16.44%)	6 (8.22%)	8 (10.96%)	4 (5.48%)	5 (6.85%)	5 (6.85%)	8 (10.96%)	4 (5.48%)	2 (2.74%)	73	-0.094	$0.149^{\dagger}$
Dizziness	4 (8.89%)	3 (6.67%)	2 (4.44%)	6 (13.33%)	1 (2.22%)	4 (8.89%)	3 (6.67%)	7 (15.56%)	10 (22.22%)	1 (2.22%)	4 (8.89%)	45	0.172	$0.003^{\dagger**}$
Disorder of consciousness	0 (0%)	3 (13.64%)	2 (9.09%)	4 (18.18%)	5 (22.73%)	5 (22.73%)	2 (9.09%)	1 (4.55%)	0 (0%)	0 (0%)	0 (0%)	22	-0.045	$0.367^{\dagger}$
Insomnia	1 (4.76%)	0 (0%)	1 (4.76%)	3 (14.29%)	1 (4.76%)	3 (14.29%)	2 (9.52%)	2 (9.52%)	6 (28.57%)	1 (4.76%)	1 (4.76%)	21	0.159	$0.009^{\dagger**}$

Data are expressed as n (%). Results are based on non-empty rows and columns in each innermost sub-table. †. Significance value of Trend Chi-Square test. *. significant at the .05 level. **. significant at the .01 level. ***. significant at the .001 level.

Table S3: Firth's penalized logistic regression analysis

Parameter	Estimate	*P* Value	OR	95%CI	
WBC in CSF (10^6 cell / L)	-0.097	0.015	0.908	-0.227	-0.012
CD3 count	0.001	0.964	1.001	-0.046	0.039
CD4 count	0.003	0.954	1.003	-0.058	0.048
CD8 count	-0.005	0.861	0.995	-0.046	0.045
PCT	0.466	0.001	1.593	0.189	0.804
ART initiation	-1.470	0.201	0.230	-6.390	0.617
Constant	-0.671	0.247	0.511	-1.736	0.429

OR: Odds Ratio. CI: confidence interval. WBC: white blood cell. CSF: Cerebrospinal fluid. PCT: procalcitonin test. ART: Antiretroviral therapy

Table S4: PCT level in two groups with cut-off value

PCT(cut-off)	Outcome		*P*-value
	Survival	Death	
PCT(0.5)			
≤0.5	174	11	<.001***
>0.5	8	7	
PCT(1.0)			
≤1.0	177	15	0.026*
>1.0	5	3	

PCT: procalcitonin test. *. significant at the .05 level. **. significant at the .01 level. ***. significant at the .001 level.

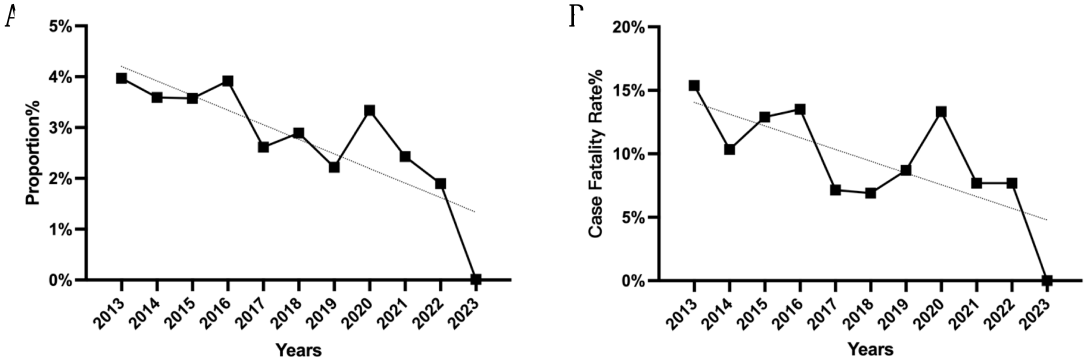

Figure S1: Baseline characters of patients with HIV-associated cryptococcal meningitis (CM). **(A)** Proportion of CM cases among HIV-infected patients **(B)** Annual cases fatality rate of CM in AIDS patients

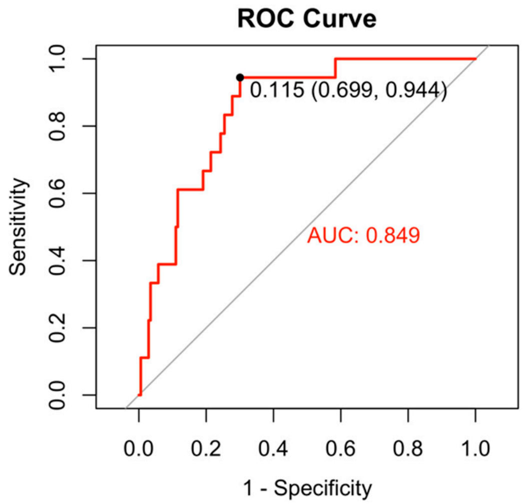

Figure S2: Receiver Operating Characteristic (ROC) curve of Table S3
